# Mechanism of 5-chloro-2-methyl-4-isothiazolin-3-one (CMIT) in controlling microbial problems in aircraft fuel systems

**DOI:** 10.1039/d3ra02970k

**Published:** 2023-06-28

**Authors:** Xiaohan Yan, Ruifang Han, Weijie Fan, Borong Shan, Jie Yang, Xiaodong Zhao

**Affiliations:** a School of Ocean, Yantai University Yantai 264005 China zhaoxiaodong23@163.com; b Qingdao Campus of Naval Aeronautical University Qingdao 266041 China wjf725@sina.cn

## Abstract

This research investigated the potential use of 5-chloro-2-methyl-4-isothiazolin-3-one (CMIT) as a biocide in aircraft fuel systems, which is rarely studied due to the unique properties of such systems. The study assessed the effectiveness of CMIT against three microbial isolates using minimum inhibitory concentrations and bacteriostatic tests, and showed that CMIT had good activity against them. Electrochemical studies were conducted to determine the impact of CMIT on the 7B04 aluminum alloy, which demonstrated that CMIT acted as a cathodic inhibitor and exhibited certain levels of short-term and long-term corrosion inhibition effects at concentrations of 100 mg L^−1^ and 60 mg L^−1^, respectively. Additionally, the research provided insights into the mechanisms governing microbial problems by studying the reaction of CMIT with glutathione and sulfate. Overall, the study suggested that CMIT may be a useful biocide in aircraft fuel systems and provided important information on its efficacy and mechanism of action.

## Abstract 2

This research investigated the potential use of 5-chloro-2-methyl-4-isothiazolin-3-one (CMIT) as a biocide in aircraft fuel systems, which is rarely studied due to the unique properties of such systems.

## Graphical abstract

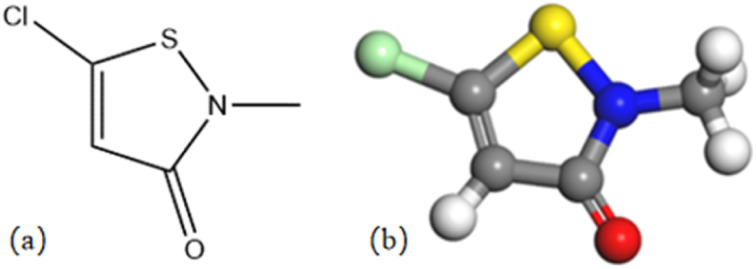

## Introduction

The 7B04 aluminum alloy has high strength, hardness, toughness and excellent processing and welding properties, and is commonly used in the manufacture of aircraft fuel systems.^1–5^ However, it is susceptible to localized corrosion during its service life,^6^ especially microbiological influenced corrosion (MIC).^7^ The structure and environment of aircraft fuel tanks determine the inevitable presence of liquid water, which readily forms a kerosene–water system that provides conditions for microbial survival. In addition, the free water, temperature, suitable pH, inorganic and organic matter in the fuel system facilitate a conducive environment for the proliferation of microorganisms, thus making it an ideal place for microbial growth.^7^

MIC is a type of corrosion caused by the attachment and activity of microorganisms on the surface of materials. Unlike traditional types of corrosion, MIC occurs spontaneously and can lead to localized corrosion such as pitting or general corrosion, depending on the metal substrate and the environment concerned.^8,9^ In fuel systems, the presence of ample nutrients can lead to widespread MIC, which can result in corrosion of fuel system materials, degradation or contamination of fuel,^9,10^ and blockage of filters and nozzles. These consequences can increase aircraft maintenance costs and affect aircraft safety.

Chemical control with biocides is a preventive treatment most frequently recommended against MIC in aircraft fuel systems.^11^ Biocides are effective in reducing the oxidation and acid production of microorganisms, and they also have an impact on extracellular proteins, extracellular polymeric substances (EPS) composition, and the expression of EPS synthesis-related genes.^12^ Despite their widespread use in various fields, only a few biocides have been adapted for use in aircraft fuel systems.^13–15^

It is CMIT that has been demonstrated to be an extremely effective biocide in the isothiazolinone class, being efficient, broad-spectrum, pollution-free, and adaptable in various fields. Isothiazolinone biocides are mainly used as preservatives in industrial production and daily life, with few cases of application in aircraft fuel systems.^16^ In addition, CMIT may also have corrosion inhibiting effects due to its unique structure.^17^ Since organic compounds containing sulfur, nitrogen, oxygen, un-saturated bonds and aromatic planar cycles are considered effective corrosion inhibitors,^18^ while their synthetic raw materials are inexpensive, the synthesis process is relatively mature, and safety is somewhat guaranteed.^19^ Therefore, this topic is of some significance for research.

## Experimental

### Materials

*L. sphaericus*, *A. lwoffii*, and *S. salmoneum* were isolated from an aircraft fuel system species in service in a marine environment. Experiments have been performed using LB culture medium for bacterial culture.

The main chemical composition of the 7B04 aluminum alloy was Ni (<0.1%), Ti (0.05–0.4%), Si (0.3%), Cu (3.2–3.7%), Zn (0.1%), Mg (2.1–2.6%), Mn (0.50–0.80%), Fe (0.3%) and aluminum residuals. Aluminum alloy specimens were sealed with epoxy resin with 1 cm^2^ surface area exposed on one side, ground with 800, 1000, 1200, and 2000 purpose sandpaper in sequence, and dried after cleaning the surface with anhydrous ethanol.

**Fig. 1:**
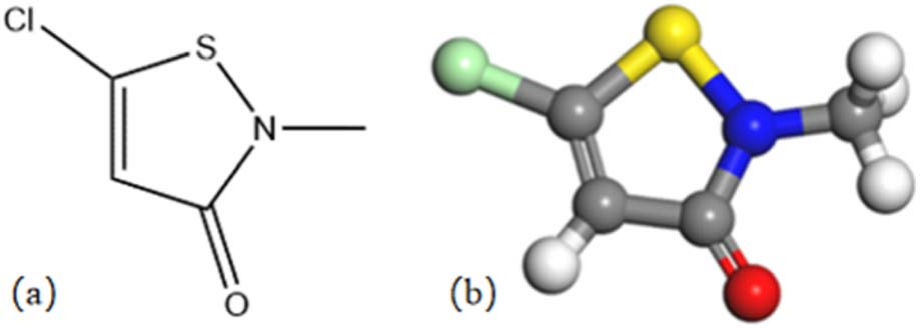
Structural formula (a) and 3D model (b) of CMIT.

CMIT is from Chemical Reagents Co. The way it is synthesized is by incremental addition of dichloromethane solution of sulfuryl chloride as an oxidizing agent to the solution of dithiodipropionamides in the same solvent at 0–10 °C.^20^ At a sulfonyl chloride to amide ratio of 3 : 1, the main products formed were 5-chloroisothiazolinone derivatives, while a ratio of 1 : 1 resulted in the formation of 5-unsubstituted analogues as the main products.^21,22^ Its structural formula and 3D model are shown in Fig. 1. The solution used for electrochemical testing was prepared from 3.5% NaCl and CMIT, which was used at concentrations ranging from 0 to 120 mg L^−1^.

### Bacteriostatic tests

The three bacterial strains were grown in a constant temperature shaker (orbital shaking) at 170 rpm for 20–48 h at 37 °C.^23–34^ The bacterial solution cultured to an absorbance of 0.8–1 A (600 nm) was diluted 1000 times with sterile dilution water. When the absorbance was between 0.8 and 1.0 (600 nm), the bacteria were generally in the middle of the exponential growth phase, during which bacterial activity was high and the accuracy of bacterial testing could be ensured. The bacterial suspension was diluted 1000 times, and 25 mL culture medium was mixed with 10 μL of bacterial suspension to poured into Petri dishes and left to condense. After the culture medium had solidified, 5 wells were punched with a 2 mm diameter punch in each Petri dish, and the 5 small wells were arranged roughly in a pentagonal shape. 10 μL of CMIT solution at different concentrations were added to each well as a control for each other. The concentration of CMIT was 0 mg L^−1^, 10 mg L^−1^, 20 mg L^−1^, 30 mg L^−1^ and 40 mg L^−1^ clockwise from the hole at the apex of the pentagon. The treated plates were incubated in a constant temperature incubator at 37 °C for 20 h. The diameter of the inhibition circle was measured to determine the sensitivity of the bacterial strains to different concentrations of CMIT.

### Minimal inhibitory concentration

10 μL of the above experimental bacterial suspension was pipetted into wells 1–11 of the 96-well plate, 85 μL of sterile liquid medium was pipetted into wells 1–10, 90 μL of sterile liquid medium was pipetted into well 11 as a positive control for normal growth of the bacterial solution, and 100 μl of sterile liquid medium was pipetted into well 12 to form a negative control with sterile growth. The CMIT was diluted twofold so that the concentrations of CMIT were 256 mg L^−1^, 128 mg L^−1^, 64 mg L^−1^, 32 mg L^−1^, 16 mg L^−1^, 8 mg L^−1^, 4 mg L^−1^, 2 mg L^−1^, 1 mg L^−1^, and 5 μL of CMIT was removed and added to wells 1–10 in descending order of concentration, with three groups of each concentration in parallel, and incubated in an incubator at 37 °C for 20 h. At the end of incubation, 10 μL of 1 mg mL^−1^ of the resazurin was added to wells 1–12 observing the color change of the resazurin.

### Electrochemical measurement

Electrochemical analysis was performed using the PARSTAT 2273 electrochemical workstation. A three-electrode system was used, with the working electrode being 7B04 aluminum alloy, the auxiliary electrode being Pt electrode, and the reference electrode being saturated KCl calomel electrode (SCE). The working electrode was immersed in the test solution for 14 days, and after the system was stabilized, the open-circuit potential was measured, and the electrochemical impedance spectroscopy was performed at the self-corrosive potential of 7B04 aluminum alloy with a sinusoidal potential amplitude of 10 mV and a frequency scan range of 10^5^ to 10^−2^ Hz. The data processing was performed using ZSimpWin software to analyze the structure of the equivalent circuit and the parameters of each component. The electrochemical polarization curve of the 7B04 aluminum alloy was tested that scanning rate was 0.33 mV s^−1^, and the scanning range was ±350 mV relative to the open circuit potential. The data was analyzed using the C-view software.

### Quantum chemical calculations

The completion of quantum chemical calculations relied on the Materials Studio software and is performed based on density functional theory (DFT). The Dmol 3 module of the software was used to optimize the molecular structure with chosen computational accuracy of Fine and PBE generalized gradient approximation. After the optimization, the CMIT molecular model was calculated, and various quantum chemical parameters were obtained, including the energy of the highest occupied molecular orbital (*E*_HOMO_), energy of the lowest unoccupied molecular orbital (*E*_LUMO_), the energy gap (Δ*E*), the electrostatic potential and the charge distribution.

## Results and discussion

### Bacteriostatic tests

**Fig. 2:**
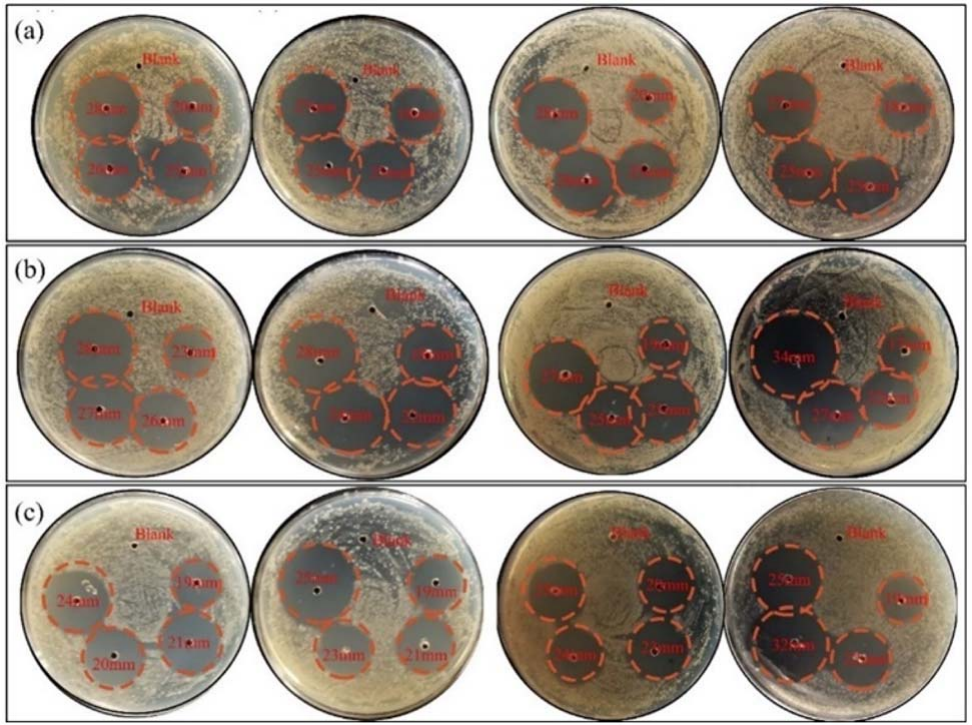
The bacteriostatic effects of different concentrations CMIT of (a) *L. sphaericus* (b) *A. lwoffii* (c) *S. salmoneum*.

**Fig. 3:**
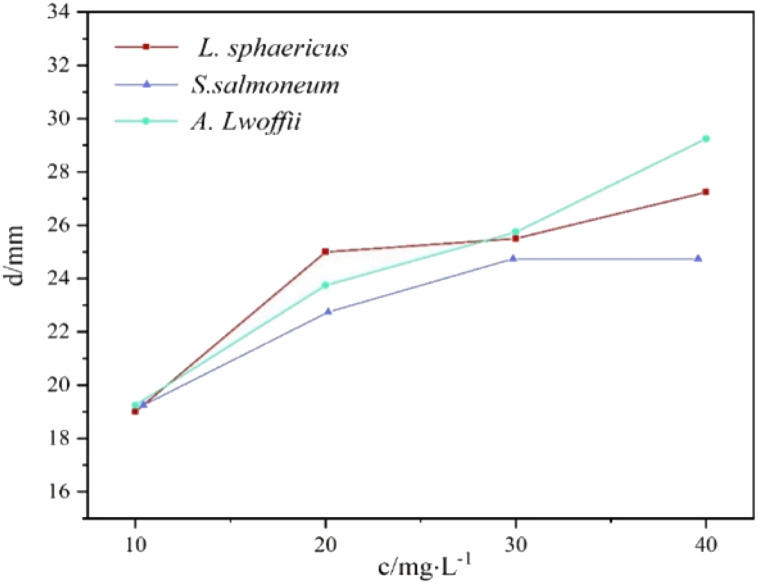
Diameters of inhibition zones produced by different concentrations of CMIT against different bacteria.

Fig. 2 and 3 illustrated the results of bacteriostatic tests conducted on three different bacteria using varying concentrations of CMIT. CMIT had a certain inhibition ability for all three bacteria. The diameter of the inhibition circle increased with increasing concentrations of CMIT for all three bacteria. The diameter difference of the inhibition circle of *L. sphaericus* was 6, 0.5 and 1.75 mm, 4.5, 2 and 3.5 mm in order for *A. lwoffii*, and 3.5, 2 and 0 mm in order for *S. salmoneum*. However, a significant decrease in the increment of the inhibition circle diameter was observed when the concentration of CMIT was equal to or greater than 30 mg L^−1^. Furthermore, Fig. 3 highlighted that CMIT demonstrated the strongest inhibitory ability against *L. sphaericus* at concentrations below 30 mg L^−1^ but the strongest inhibitory ability against *A. lwoffii* at concentrations above 30 mg L^−1^. It was observed that the inhibitory ability of CMIT was significantly smaller for *S. salmoneum* than for *L. sphaericus* and *A. lwoffii*.

At a concentration of 10 mg L^−1^, the diameter of the inhibition zone of the three bacteria was generally. This was because the bacteria showed some level of resistance to CMIT, and the low concentration of CMIT was insufficient to highlight the difference in resistance among the three bacteria. However, as the concentration of CMIT increased, the diameter of inhibition zones for the three bacteria differed greatly, indicating varying levels of resistance to CMIT.

*S. salmoneum* showed the weakest response to CMIT inhibition. This was because *S. salmoneum* has flagella and locomotive behavior that can facilitate the formation of biofilms.^35^ Studies by Lubarsky and Périamé had shown that bacteria with greater resistance to CMIT had a higher ability to form biofilms.^36,37^ Compared to *L. sphaericus* and *A. lwoffii*, which lacked locomotion, CMIT had the weakest inhibitory effect on *S. salmoneum*.

CMIT generally has stronger inhibition ability against Gram-positive bacteria.^21^*L. sphaericus* is a Gram-positive bacterium, while *A. lwoffii* is a Gram-negative bacterium. This explained why *L. sphaericus* had a larger antibacterial zone diameter at a concentration of 20 mg L^−1^. However, as the concentration increased, the inhibitory effect on *A. lwoffii* gradually exceeded that of *L. sphaericus*. The mechanism of action of CMIT involved intracellular access by diffusing through the cell membrane of bacteria.^38^ As the concentration of CMIT increased, the diffusion of CMIT was limited by the monolayer surrounded by a thick peptidoglycan found in Gram-positive bacteria.^39^ The difference in the diameter of the inhibition zone between the two bacteria was therefore mainly due to the diffusion of CMIT.

### Minimum inhibitory concentration

**Fig. 4:**
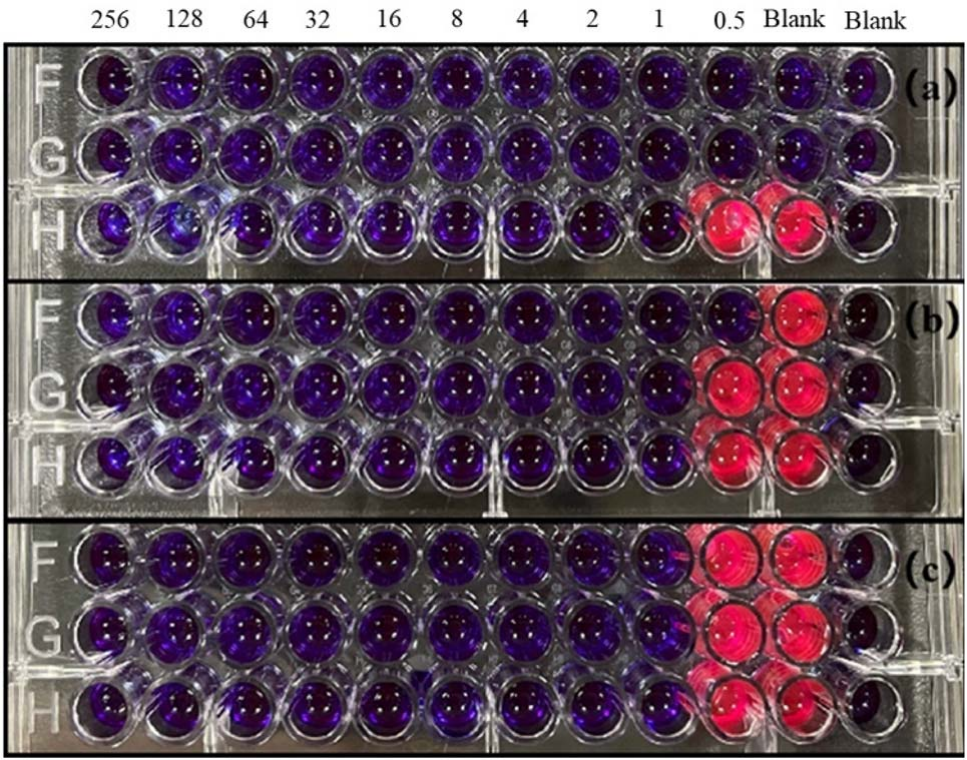
Minimum inhibitory concentration results of CMIT (a) *S. salmoneum*, (b) *L. sphaericus*, (c) *A. lwoffii*.

The minimum inhibitory concentration of the CMIT was determined using resazurin to indicate. If the CMIT concentration is sufficient to kill the bacteria, resazurin will appear blue, otherwise it will appear pink or white. Thus, the color change could be used to determine whether CMIT can act as a fungicide. It could be seen that well 12 of the negative control with 100 μl of sterile medium in Fig. 4 was blue, indicating that the culture medium was indeed sterile, which provided support for the reliability of the experiment. The positive controls of *L. sphaericus* and *A. lwoffii* were pink, indicating that both bacteria-maintained growth in this culture medium and grew faster. While only one group of positive controls of *S. salmoneum* showed pink color, which may be due to its activity was low and growth rate was slow, making resazurin did not play a chromogenic role. Meanwhile, Fig. 4 pointed out that the minimum inhibitory concentration of CMIT was 1 mg L^−1^ for all three bacteria.

The minimum inhibitory concentration (MIC) of CMIT did not exhibit any significant differences in the inhibition of the three bacterial strains. This could be because CMIT had fuller contact with the bacteria in the bacterial suspension during the experiment, as compared to the bacteriostatic tests. The role of CMIT may depend on exposure time. It is speculated that the biological activity of CMIT arises from their interaction with intracellular sulfur-containing proteins, enzymes, or simple molecules such as glutathione, which leads to ring-opening and disulfide bond formation and ultimately impairs cellular function.^25,40–44^ The bioactivity of isothiazolinones is known to originate form intracellular access by diffusing through the cell membrane of bacteria or the cell wall of fungi.^38^ The formation of cell access by CMIT depends on it having sufficient contact time with bacteria, indicating that CMIT requires a certain amount of time to prepare before entering the cell and exerting its effects. Upon entering the cell, the electron-deficient sulfur atom of the CMIT moiety can react with cellular components that contain nucleophilic groups, such as the thiol moieties of cysteine units, gradually impairing their activity and leading to cell death.^38,45^ CMIT initially inhibits bacterial metabolism and growth, eventually leading to irreversible loss of bacterial activity.^46^ The efficacy of this process depends on the duration of exposure to CMIT within the cells. Despite its time-dependent action, CMIT's mechanism of action allows it to sustainably perform its role, resulting in time-dependent inhibition of various interfering bacteria that can last up to 48 hours.

### Electrochemical impedance spectroscopy

**Fig. 5:**
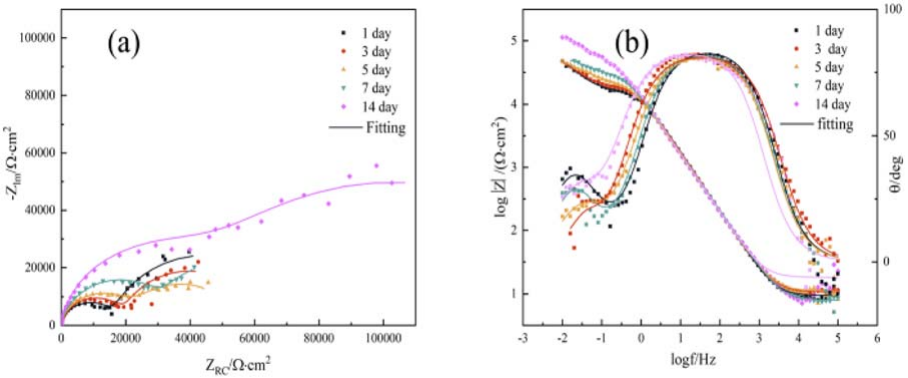
(a) Nyquist and (b) Bode plots of 7B04 aluminum alloy immersed in 3.5% NaCl for different times.

The effect of CMIT on the corrosion behavior of 7B04 aluminum alloy in terms of electrochemistry was further investigated using electrochemical impedance spectroscopy.^47^Fig. 5 showed the Nyquist and Bode plots of 7B04 aluminum alloy specimens immersed in 3.5% NaCl solution for different times.

The Nyquist plot showed a capacitance-like loop, which appeared as two time constants for the 7B04 aluminum alloy system. The first time constant corresponded to the double layer capacitance related to charge transfer,^48^ while the second time constant, which was not well-defined at intermediate frequencies, may indicate another Faraday process at the metal–electrolyte interface, such as the adsorption of another intermediate product (possibly a cation) thus forming an additional circuit,^49,50^ the diameter of the capacitive loop was proportional to the transfer resistance *R*_ct_ of the charge on the electrode surface, and its extreme value was observed at 14 days. In general, the larger the *R*_ct_, the smaller the corrosion rate of the electrode. The formation of an oxide film on the surface of the aluminum alloy could explain the reduction in its corrosion rate, leading to larger values of *R*_ct_.

**Fig. 6:**
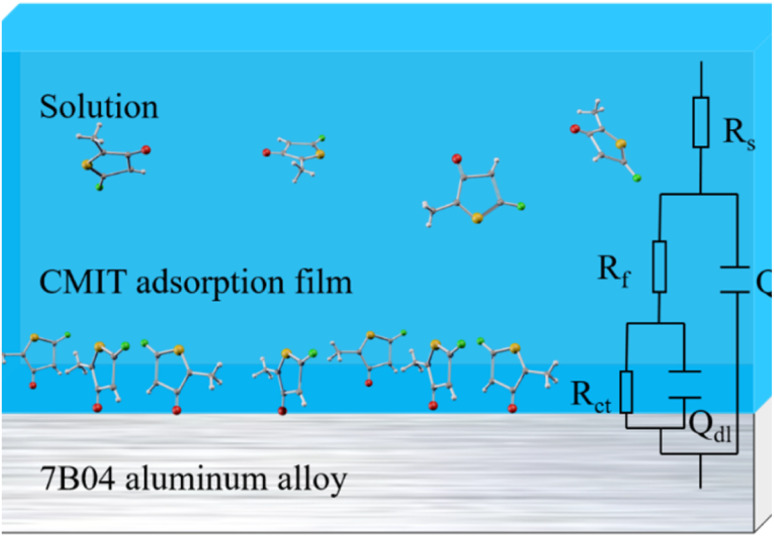
The equivalent electrical circuit used to fit the EIS experimental data for 7B04 aluminum alloy.

The data presented in Table 1 were obtained by fitting electrochemical impedance spectroscopy results to the standard circuit depicted in Fig. 6. The dataset included information on various parameters such as the solution resistance (*R*_s_), the oxide film resistance (*R*_f_), the charge transfer resistance (*R*_ct_), the double layer capacitance (*Q*_dl_), and the film capacitance of the corrosion products on the aluminum alloy surface (*Q*_f_). At high frequencies, the intercept corresponded to the solution resistance (*R*_s_), while at low frequencies, it corresponded to (*R*_s_ + *R*_ct_). The difference between these two values provided the charge transfer resistance (*R*_ct_), which was a measure of the electron current through the surface and was inversely proportional to the corrosion rate.^51^

**Table 1:** Fitting electrochemical parameters of 7B04 aluminum alloy after immersion in 3.5% NaCl

Days	*R*_s_ (W cm^2^)	*Q*_f_ (mF cm^−2^)	*Q*_dl_ (mF cm^−2^)	*R*_f_ (W cm^−2^)	*R*_ct_ (W cm^−2^)
1	9.432	1.112 × 10^−5^	2.18 × 10^−4^	1.701 × 10^4^	5.821 × 10^4^
3	10.87	1.183 × 10^−5^	2.791 × 10^−4^	2.092 × 10^4^	4.036 × 10^4^
5	11.16	1.322 × 10^−5^	2.099 × 10^−4^	2.446 × 10^4^	3.089 × 10^4^
7	7.925	1.312 × 10^−5^	2.892 × 10^−4^	3.556 × 10^4^	3.692 × 10^4^
14	17.92	1.248 × 10^−5^	8.255 × 10^−5^	5.944 × 10^4^	1.085 × 10^5^

Table 1 displayed a trend in the values of *R*_ct_, which showed a decrease followed by an increase with a peak value occurring at 14 days. This indicated that an oxide film was gradually forming on the surface of the aluminum alloy during the corrosion process. The oxide film acted as a protective layer, slowing down the corrosion process. The *Q*_dl_ value at 14 days was 8.255 × 10^−5^, which was smaller than at other times, suggesting a decrease in the local dielectric constant or an increase in the thickness of the double layer capacitance.^23,31^ This also indicated the formation of an oxide film on the surface of the aluminum alloy. Moreover, *R*_s_ at 14 days was much greater than at other times, indicating a decrease in the conductivity of the solution due to the formation of oxide films on the surface of the aluminum alloy and the adsorption of corrosion products. This reduced the concentration of conductive ions in the solution. It was important to note that the magnitude of *R*_s_ was influenced by factors such as ion concentration and type, as well as temperature.^8^

The electrochemical impedance spectroscopy of 7B04 aluminum alloy immersed in 3.5% NaCl solution with varying concentrations of CMIT was analysed using the standard circuit shown in Fig. 6 for a period of 14 days. The resulting electrochemical kinetic parameters, including the solution resistance (*R*_s_), aluminum alloy surface corrosion product film capacitance (*Q*_f_), oxide film resistance (*R*_f_), double layer capacitance (*Q*_dl_), and charge transfer resistance (*R*_ct_) with different CMIT concentrations, was presented in Table 2.

**Table 2:** Fitting electrochemical parameters of 7B04 aluminum alloy after immersion in different concentrations of CMIT for different times

Days	*C*_CMIT_ (mg L^−1^)	*R*_s_ (W cm^2^)	*Q*_f_ (mF cm^−2^)	*R*_f_ (W cm^−2^)	*Q*_dl_ (mF cm^−2^)	*R*_ct_ (W cm^−2^)	*E*_i_ (%)
1	40	9.478	1.185 × 10^−5^	2.489 × 10^4^	1.53 × 10^−4^	1.404 × 10^5^	58.54
	60	11.46	1.151 × 10^−5^	2.707 × 10^4^	1.167 × 10^−4^	1.486 × 10^5^	60.83
	80	11.71	8.772 × 10^−6^	1.401 × 10^4^	1.819 × 10^−4^	2.198 × 10^4^	—
	100	23.19	1.158 × 10^−5^	4.111 × 10^4^	1.167 × 10^−4^	1.593 × 10^5^	63.46
	120	9.477	1.214 × 10^−5^	2.459 × 10^4^	1.78 × 10^−4^	9.354 × 10^4^	37.77
3	40	6.914	1.275 × 10^−5^	2.489 × 10^4^	2.003 × 10^−4^	5.398 × 10^4^	25.24
	60	41.98	1.187 × 10^−5^	2.707 × 10^4^	1.175 × 10^−4^	1.186 × 10^5^	65.97
	80	8.435	1.229 × 10^−5^	1.401 × 10^4^	1.992 × 10^−4^	5.623 × 10^4^	28.22
	100	272.8	8.852 × 10^−6^	4.111 × 10^4^	7.4 × 10^−5^	2.242 × 10^5^	82.00
	120	8.392	1.334 × 10^−5^	2.459 × 10^4^	2.639 × 10^−4^	9.96 × 10^4^	59.48
5	40	788.8	1.271 × 10^−5^	2.084 × 10^4^	9.625 × 10^−5^	9.254 × 10^4^	66.62
	60	14.95	1.242 × 10^−5^	3.887 × 10^4^	1.102 × 10^−4^	1.431 × 10^5^	78.41
	80	11.41	9.835 × 10^−6^	49.5	4.112 × 10^−6^	3.587 × 10^4^	13.88
	100	11.1	1.283 × 10^−5^	7.649 × 10^4^	7.285 × 10^−5^	1.834 × 10^5^	83.16
	120	12.03	1.196 × 10^−5^	3.419 × 10^4^	1.411 × 10^−4^	1.53 × 10^5^	79.81
7	40	7.222	1.334 × 10^−5^	4.192 × 10^4^	2.22 × 10^−4^	5.871 × 10^4^	37.11
	60	401.9	9.44 × 10^−6^	2.611 × 10^4^	1.151 × 10^−4^	1.126 × 10^5^	67.21
	80	10.04	1.4 × 10^−5^	4.693 × 10^4^	2.47 × 10^−4^	4.792 × 10^4^	22.95
14	60	11.1	1.283 × 10^−5^	7.649 × 10^4^	7.285 × 10^−5^	1.834 × 10^5^	40.84
	80	12.22	1.439 × 10^−5^	9.356 × 10^4^	1.125 × 10^−4^	1.586 × 10^5^	31.59

Table 2 presented the electrochemical data of the CMIT system with corrosion inhibition at 14 days, as compared to Table 1. The table was screened to obtain the relevant data. The corrosion inhibition efficiency (*E*_i_) was calculated from the electrochemical impedance spectroscopy using the following equation:
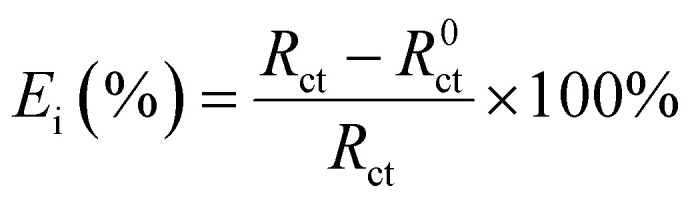
Here, *R*^0^_ct_ and *R*_ct_ are the charge transfer resistance values in the absence and presence of inhibitors, respectively.

As was seen from Table 2, almost all concentrations of the CMIT system exhibited corrosion inhibition in the first 5 days of immersion. However, the corrosion inhibition efficiency did not have a good linear relationship with the change in CMIT concentration. The CMIT solution system with a concentration of 80 mg L^−1^ had low corrosion inhibition efficiency of 0%, 28.22%, and 13.88%, indicating poor corrosion inhibition. The corrosion inhibition efficiency of the CMIT solution increased and then decreased with its concentration, which was related to the covering effect of CMIT on the metal surface at different concentrations.

The 100 mg per L CMIT solution showed good corrosion inhibition efficiency, with values of 63.46%, 82.00%, and 83.16% at immersion times of 1, 3, and 5 days, respectively. This was much greater than the corrosion inhibition efficiency of other systems with the same immersion time, indicating good corrosion inhibition. However, the higher concentration of 120 mg per L CMIT solution system did not exhibit good corrosion inhibition efficiency, with values of 37.77%, 59.48%, and 79.81%. Increasing the concentration of the CMIT solution beyond 100 mg L^−1^ did not improve its corrosion inhibition efficiency.

The 100 mg per L CMIT solution presented good corrosion inhibition efficiency, with values of 63.46%, 82.00%, and 83.16% at immersion times of 1, 3, and 5 days, respectively. This was much greater than the corrosion inhibition efficiency of other systems with the same immersion time, indicating good corrosion inhibition. However, the higher concentration of 120 mg per L CMIT solution system did not exhibit good corrosion inhibition efficiency, with values of 37.77%, 59.48%, and 79.81%. Increasing the concentration of the CMIT solution beyond 100 mg L^−1^ did not improve its corrosion inhibition efficiency.

When the immersion time reached 7 and 14 days, the 100 mg per L and 120 mg per L CMIT solution systems lost their corrosion inhibition effect. However, the 60 mg per L CMIT solution exhibited corrosion inhibition for 14 days, with a corrosion inhibition efficiency of 60.83%, 65.97%, 78.41%, 67.21%, and 40.84%, respectively. This suggested that a CMIT solution concentration of 60 mg L^−1^ had a longer corrosion inhibition effect.

**Fig. 7:**
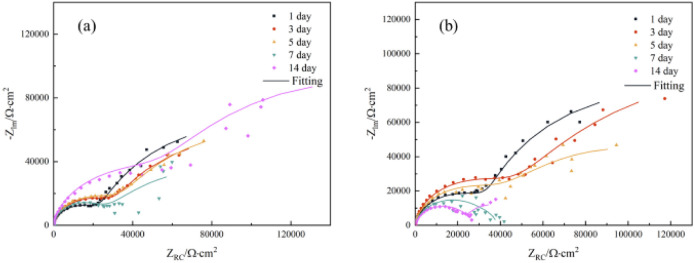
Nyquist plots of 7B04 aluminum alloy immersed in CMIT with the different times for different concentrations ((a) 60 mg L^−1^ (b) 100 mg L^−1^).

Fig. 7 displayed the impedance spectroscopy of the CMIT solutions with concentrations of 60 mg L^−1^ and 100 mg L^−1^. The following results were obtained when compared with the blank control in Fig. 5: (i) the impedance spectroscopy before and after the addition of CMIT had almost similar shapes, indicating that the CMIT did not change the corrosion reaction mechanism.^52–54^ (ii) The obtained impedance spectrogram curves showed an inhibited semicircle (loop capacitance). This semicircle depression was related to the inhomogeneity of the metal surface, roughness, and distribution of active sites.^55,56^ The appearance of the second time constant of the loop capacitance may be related to the adsorption of corrosion products and CMIT on the metal surface.^49,50^ As seen in Fig. 7(b), the diameter of the capacitive loop at 7 and 14 days, when the corrosion inhibition was lost, was much smaller than the other times for a CMIT concentration of 100 mg L^−1^.

Based on the data in Table 3, it was observed that the system with higher corrosion inhibition efficiency had a much lower *Q*_dl_ than other systems. Furthermore, it could be seen that the system with the maximum corrosion inhibition efficiency at a particular immersion time generally exhibited the minimum *Q*_dl_ value during that immersion time. This indicated a negative correlation between *Q*_dl_ and corrosion inhibition efficiency, which was usually observed in protected systems. The inhibition was attributed to the formation of an adsorbed surface film by the adsorption inhibitor on the metal surface. This film restricted access of charged particles to the metal surface, causing a decrease in *Q*_dl_.

**Table 3:** Electrochemical parameters obtained by polarization curve fitting

CMIT (mg L^−1^)	*E*_corr_ (mV)	*I*_corr_ (μA cm^−2^)	*B*_a_ (mV dec^−1^)	*B*_c_ (mV dec^−1^)	*E*_i_ (%)
Blank	−1151.9	1.2682	273.47	−78.372	—
40	−1022.5	1.9744	503.24	−131.93	—
60	−1210.0	0.5427	122.19	−51.798	57.21
80	−1267.7	0.7278	68.379	−77.461	42.61
100	−1136.3	2.7861	279.52	−102.48	—
120	−1262.6	1.5251	258.36	−132.06	—

### Potentiodynamic polarization curves

**Fig. 8:**
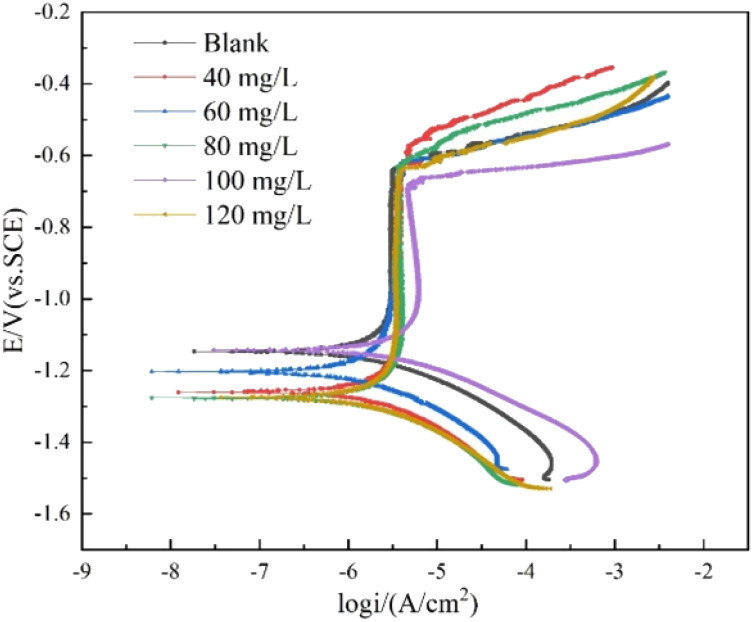
Polarization curves of 7B04 aluminum alloy immersed in different systems.

Fig. 8 depicted the potentiodynamic polarization curves of 7B04 aluminum alloy immersed in 3.5% NaCl solution with different concentrations of CMIT for 14 days. Table 4 presented the variation of electrochemical kinetic parameters, such as corrosion potential (*E*_corr_), corrosion current density (*I*_corr_), and corrosion inhibition efficiency (*E*_i_) with CMIT concentration. *E*_i_ was calculated from the polarization measurements using the following equation:
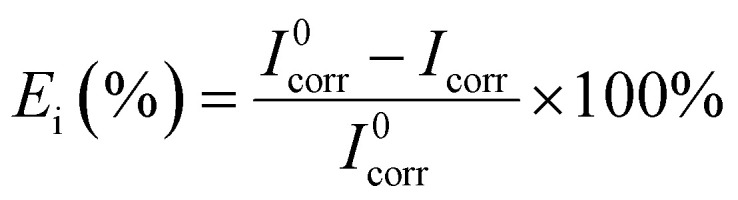
here, *I*^0^_corr_ and *I*_corr_ are the corrosion current densities in the absence and presence of inhibitors, respectively.

**Table 4:** Chemical structural descriptors of CMIT

Molecular	*E*_HOMO_/eV	*E*_LUMO_/eV	Δ*E*/eV
CMIT	−0.0699	0.0612	0.131

The results shown in Fig. 8 and Table 3 demonstrated several key findings. Firstly, the addition of CMIT caused *E*_corr_ values to shift towards more negative potentials, with a maximum shift exceeding 85 mV. This shift may be attributed to the decrease in cathodic reaction rates, suggesting that CMIT behaved as a cathodic inhibitor. Secondly, the Tafel slopes of both anode and cathode were only slightly shifted, indicating that the electrochemical mechanism remained unchanged in the presence of CMIT. Finally, after the addition of CMIT, the *I*_corr_ decreased at CMIT concentrations of 60 mg L^−1^ and 80 mg L^−1^ compared to the blank, with corrosion inhibition efficiency of 57.21% and 42.61%, respectively. These results are consistent with the findings of electrochemical impedance spectroscopy.

### Quantum chemical calculations

**Fig. 9:**
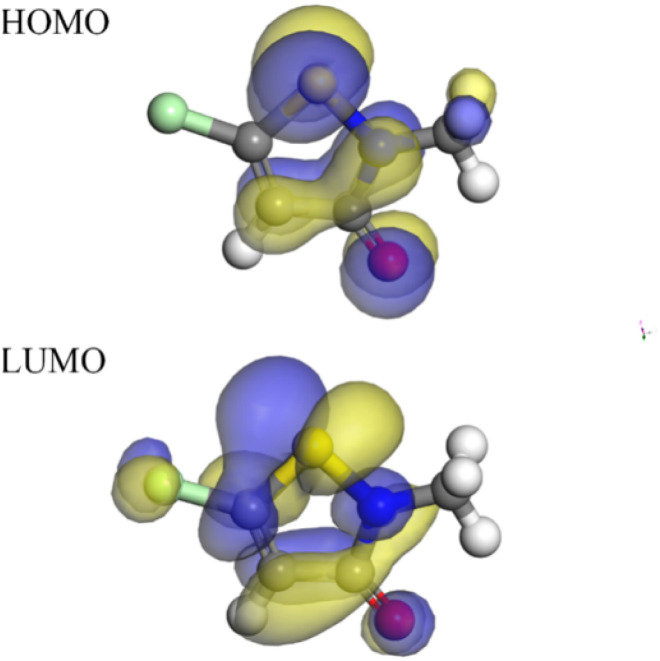
HOMO density distribution and the LUMO density distribution for CMIT molecule obtained with DFT.

Quantum chemical calculations could offer valuable insights into the mechanism of action of CMIT at the molecular level. Density functional theory was a widely accepted and extensively used theory in the field of corrosion inhibition mechanism exploration. It had been demonstrated that the electronic distribution and geometric structure within the corrosion inhibitor molecule played a crucial role in determining its corrosion inhibition properties.^57^ In this study, the experimental findings on the corrosion inhibition efficiency of CMIT were correlated with its structural and electronic properties to obtain the *E*_HOMO_ and *E*_LUMO_ electron density distributions of CMIT molecules, as depicted in Fig. 9.

**Fig. 10:**
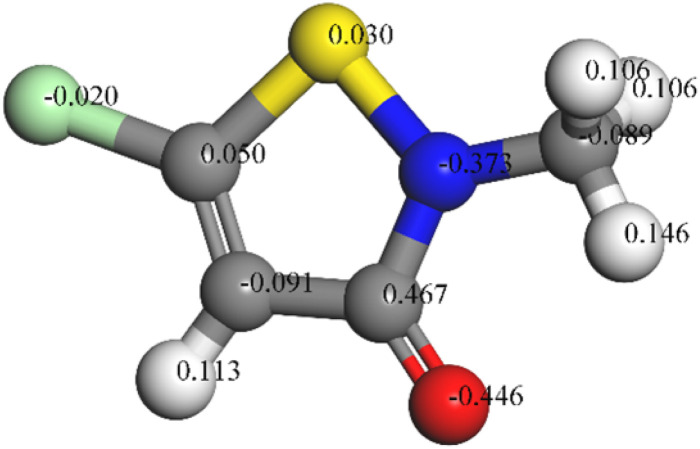
Mulliken atomic charges calculated for CMIT molecule.

The chemical reactivity was determined by the interaction between the *E*_HOMO_ and *E*_LUMO_ levels of the reactants. As illustrated in Fig. 10, the CMIT molecule had a dense charge distribution in the sulfur atom, oxygen atom, and carbon atoms 2, 3, and 4 of the five-membered ring *E*_HOMO_, with a small amount of charge at the methyl group. Additionally, the *E*_LUMO_ charge distribution was equally dense in the regions of chlorine atom, sulfur atom, oxygen atom, and carbon atoms 2, 3, 4, and 5 of the five-membered ring. This suggested that the five-membered ring, nitrogen atoms, and sulfur atoms may be the active part of the CMIT molecule. The electron density analysis of *E*_HOMO_ and *E*_LUMO_ revealed that the CMIT molecule had the ability to donate and accept electrons under favorable conditions, which was the primary reason for the adsorption of CMIT on the aluminum alloy surface.

Δ*E* was the energy difference between the HOMO and LUMO orbitals, and it represented the interaction ability of CMIT with the metal. A smaller value of Δ*E* indicated that CMIT could be adsorbed on the metal surface more easily.^57^ In this study, the value of Δ*E* was 0.131 eV, indicating that CMIT has a good adsorption capacity.

**Fig. 11:**
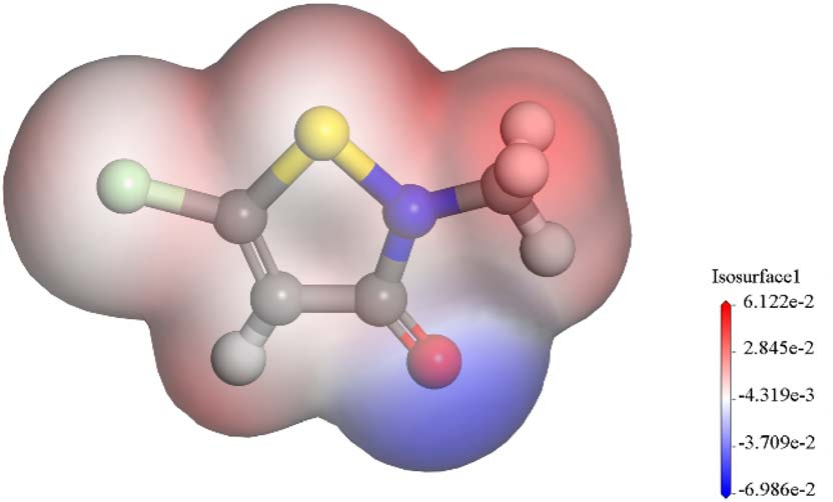
The MEP surfaces of the CMIT.

The molecular electrostatic potential (MEP) was a crucial component of mapping molecular surfaces. It helped predict the most active position on the molecular surface based on the distribution of charges and electrons. When molecules were in proximity, the MEP played a critical role in determining the mode of interaction. Fig. 11 illustrated the MEP of a CMIT molecule, with the highest MEP value located at the oxygen atom. Combined with the above analysis, the oxygen atom was the active center for adsorption on the metal surface.

### CMIT sterilization mechanism

Biocides are substances able to destroy, inhibit, and control harmful organisms.^58^ Isothiazolone biocides, in particular, are highly effective in inhibiting microbial growth^59^ and are commonly used in household cleaning and personal care products,^60^ biodiesel,^61^ cooling water systems,^62^ food packaging,^63^ and water-based paint antifouling,^64,65^ with little use in aircraft fuel and fuel tank systems. However, due to the hygroscopic nature of aircraft fuel and the unique structure of fuel tank systems, the entire aircraft fuel system is highly susceptible to microbial contamination.^66^ In the presence of water, oil-like components provide an ideal environment for microbial growth, leading to their rapid proliferation in the aircraft fuel system.^61^ This can cause two major problems: MIC and microbial contamination,^67^ which can impact the properties of 7B04 aluminum alloy and degrade the performance of aircraft fuel.

CMIT was required to effectively inhibit microbial growth in the fuel system without causing corrosion to the 7B04 aluminum alloy. The mechanism of action of CMIT primarily involves rapid inhibition of growth and metabolism (within minutes) followed by irreversible cellular damage leading to loss of viability (within hours). CMIT works by disrupting the metabolic pathways of cellular dehydrogenases, inhibiting key physiological functions of microorganisms such as growth, respiration (oxygen consumption), and energy production (ATP synthesis).^68^ CMIT reacts with glutathione (GSH),^58^ which is an important intracellular antioxidant and regulator of oxidation–reduction potential. GSH and its related enzymes play a crucial role in protecting cells from free radicals, peroxides, and toxins.^69,70^ Its reduced form is a ubiquitous nucleophilic reagent that converts various electrophilic substances under physiological conditions. GSH-dependent enzymes significantly facilitate most chemical reactions in many metabolic pathways.^71^ A decrease in GSH content can be a potential signal for early activation of apoptosis, and the subsequent production of oxygen radicals drives apoptosis.^72^ The reaction of CMIT with the sulfhydryl, which is the reactive group on GSH,^73^ renders it inactive and eventually leads to cellular inactivation.

Histone acetylation and deacetylation play a crucial role in gene transcription and cell cycle progression. CMIT can inhibit histone acetyltransferase p300/CBP-associated factor (PCAF), and its inhibitory effect on PCAF may arise from its action on thiols, which are the active site of the related enzyme.^74^ Although CMIT does not direct react with thiols, their reaction with sulfate is fast.^58^ The reaction of CMIT with sulfate includes a nucleophilic attack on the sulfur atom accompanied by the cleavage of the S–N bond, resulting in the generation of a ring-opening product that can be further reacted with, ultimately producing a variety of products.^74^ These products affect the activity of the relevant enzyme and lead to cellular inactivation. Additionally, the growth inhibition pattern and morphological changes induced by CMIT suggest that the compound also inhibits the initiation of DNA replication,^75^ thus inhibiting microbial activity.

Additionally, the activity of isothiazolone compounds can be affected by the presence of different substituents. The activity of 3(2*H*)-isothiazolones substituted at the 5-position of the isothiazolone ring with chloro was higher or equal when compared to unsubstituted analogous compounds.^22^ For instance, 5 unsubstituted isothiazolones (MIT) were less effective than C5 chloro-substituted (CMIT) analogues. It may be explained by the fact that CMIT is believed to more easily moved towards the innermost structures of bacterial cells compared to MIT.^76^ Because the C5 position and the S–N in CMIT are both susceptible to nucleophilic attack, whereas in MIT, only the S–N is vulnerable to this type of attack.^77^ Furthermore, the majority of isothiazolinone 2-position substitutes exhibited higher bactericidal activity against Gram-positive bacteria.^21^ It is possible that an additional mode of action is responsible for the exceptional potency of this compound. However, when isothiazolinone 2-position is substituted with the appropriate phenyl, the resulting compounds exhibit higher activity against Gram-negative bacteria. This finding is consistent with the well-known tendency of Gram-negative bacteria to be more susceptible to hydrophilic compounds, whereas lipophilic antibiotics tend to be ineffective against these organisms.^21,78^ The antibacterial activity of isothiazolinone substitution is determined by a combination of several factors, including electronic properties, size, and hydrophobicity.

CMIT has a destructive effect on bacterial biofilms,^79^ which are a large number of mutually adherent cell populations adsorbed on the surface of 7B04 aluminum alloys. Biofilms can promote material corrosion or slow it down, depending on the concentration, adsorption, charge, and 3D structure of different microbial EPS components,^80^ Therefore, CMIT has the potential to slow down MIC. In addition, CMIT did not negatively affect the 7B04 aluminum alloy and had a corrosion inhibition effect on the 7B04 aluminum alloy for the entire experimental period (14 days) when the CMIT concentration was less than 100 mg L^−1^. Even at CMIT concentrations of 100 mg L^−1^ and 120 mg L^−1^ for a short period (5 days), the alloy was not adversely affected.

The corrosion process of aluminum alloys is a complex phenomenon that involves the interaction between ions in a corrosive medium and the surface of the aluminum alloy. The dissolution–precipitation mechanism of corrosion of aluminum alloys at the anodic position in aqueous chloride solutions occurs through several steps. According to Pourbaix map, at 25 °C, aluminum passivates (protected by oxide film) in aqueous solutions with pH 4–8.5.^81^ However, the presence of reactive chloride ions in a NaCl solution prevents the formation of this passivation film on the aluminum alloy surface and accelerates the anodic dissolution process. Pitting corrosion occurs due to the migration of chloride ions through the oxide film forming an oxide–chlorine complex or simply AlCl_3_.^82^ The AlCl_3_ further reacts with chloride ions to form highly soluble AlCl_4_-complexes, which diffuse into the main body of the solution and provide space for corrosion to continue to occur. Additionally, the complex formed by Al^3+^ and Cl^−^, upon reaction with water or hydroxyl groups, precipitates as aluminum hydroxide.^83^ Meanwhile, Cl^−^ is released from the complex and returns to the solution to continue the dissolution–precipitation reaction, making the process autocatalytic in nature.^81^

CMIT acts as a corrosion inhibitor by protecting the metal surface from the reaction of the medium through the adsorption of its polar groups on the active sites on the metal surface.^84,85^

The adsorption of CMIT molecules on the metal surface leads to the formation of an arranged adsorption layer at the metal/liquid interface, which decreases the dissolution reaction of the metal. Increasing the concentration of inhibitor in the medium increases the number of adsorbed molecules on the metal surface, thereby increasing the thickness of the inhibitor layer. The dense adsorbed inhibitor layer maintains a higher isolation of the metal surface and therefore provides higher protection.^81,86,87^

## Conclusions

(1) CMIT has a significant inhibitory effect on *L. sphaericus*, *A. lwoffii*, and *S. salmoneum*, with a minimum inhibitory concentration of 1 mg L^−1^ for all of them. However, the inhibitory ability is weaker for *S. salmoneum*.

(2) CMIT does not aggravate corrosion of the 7B04 aluminum alloy and exhibits a certain level of corrosion inhibition. At a concentration of 100 mg L^−1^, it shows good corrosion inhibition efficiency in the short term (5 days), while a concentration of 60 mg L^−1^ has a longer-term corrosion inhibition effect.

(3) The primary way CMIT sterilizes is by reacting with GSH (a naturally occurring molecule in cells) and inhibiting PCAF (an enzyme involved in gene regulation) through a rapid reaction with sulfate. By interfering with these cellular processes, CMIT disrupts bacterial function and ultimately leads to their death.

## Conflicts of interest

There are no conflicts to declare.

## Supplementary Material
